# A120 AUTOMATED BOWEL PREPARATION DETECTION WITH DEEP. CONVOLUTIONAL NEURAL NETWORKS

**DOI:** 10.1093/jcag/gwab049.119

**Published:** 2022-02-21

**Authors:** D J Low, Z Hong, A Mukherjee, S Jugnundan, S Grover

**Affiliations:** 1 Internal Medicine, University of Toronto, Toronto, ON, Canada; 2 Massachusetts Institute of Technology, Cambridge, MA; 3 Indian Institute of Engineering Science and Technology, Howrah, West Bengal, India

## Abstract

**Background:**

Introduction: Bowel preparation inadequacy has been shown to increase post-colonoscopy colorectal cancer. As such, the USMSTF recommends repeating colonoscopy within 1 year if bowel preparation is inadequate. However, bowel preparation documentation is variable in clinical practice, and physician recommendations adherent to USMTF guidelines are inconsistent.

**Aims:**

Aims: We present an automated computer assisted method using deep convolutional neural networks to detect bowel preparation and adequacy of bowel preparation with the Boston Bowel Preparation Scale (BBPS).

**Methods:**

Methods: We extracted 38523 images of colonic lumen between 2015 and 2017 from screening colonoscopies. Bowel preparation scores were assessed with BBPS. Adequate bowel preparation was defined as BBPS ≥2, and inadequate bowel preparation was defined as BBPS <2. The dataset was split into 26966 images for training, 7704 for validation, and 3853 for testing. Training data was sampled with replacement from a multinomial distribution to balance subclass distributions in each batch. We developed 2 convoluted neural networks (CNN) using PyTorch with a Densenet-169 backbone pre-trained on ImageNet and fine-tuned on our data for classifying adequacy of bowel preparation (binary) and for subclassification of BBPS (multi-class). We used Adam optimiser with an initial learning rate of 3x10^-4^ and a scheduler to decay the learning rate of each parameter group by 0.1 every 7 epochs along with focal loss as our criterion for both classifiers.

**Results:**

Results: The overall accuracy on the test data set for BBPS subclassification was 0.91. The sensitivity for BBPS 0, 1, 2 and 3 were 0.84, 0.91, 0.86, and 0.96, respectively. The specificity for BBPS 0, 1, 2, and 3 were 1.00, 0.98, 0.95, and 0.93, respectively. The overall accuracy of the test data set for adequacy of bowel preparation was 0.97. The sensitivity for adequacy of bowel preparation for BBPS <2 and BBPS ≥2 was 0.92 and 0.99, respectively. The specificity for adequacy of bowel preparation for BBPS <2 and BBPS ≥2 was 0.99 and 0.92, respectively.

**Conclusions:**

**Conclusion:** We present an automated computer-assisted detection method of bowel preparation with deep convolutional neural networks. The algorithm is capable of accurate classification of adequacy of bowel preparation (97%) and subclassification of bowel preparation (91%) with high sensitivity and specificity. This algorithm can be applied to automate documentation of bowel preparation and adequacy of bowel preparation. Additional studies will need to be conducted to demonstrate its applicability in real-time colonoscopy.

**Funding Agencies:**

None

